# Infants with Teeeth at Birth

**Published:** 1862-12

**Authors:** Geo. T. Elliott

**Affiliations:** Physician to the Bellevue Hospital


From the American Medical Times.

INFANTS WITH TEETH AT BIRTH.—*Sir:*—In the lying-in wards of Bellevue there are now two infants under my care who were born with teeth, viz: William Hoffman, sixth child, weight at birth six and one-half pounds, puny, fully developed; right middle incisor in lower jaw well formed and protruded, but placed athwart the jaw.

Annie Morse, first child, weighed at birth seven pounds, fully developed; two middle incissors in the lower jaw, both well formed, but loose; right incisor set obliquely in the alveolar process. Yours, etc.,

GEO. T. ELLIOTT, JR.
*Physician to the Bellevue Hospital.*

